# Untargeted LC/MS-Based Metabolic Phenotyping of Hypopituitarism in Young Males

**DOI:** 10.3389/fphar.2021.684869

**Published:** 2021-07-08

**Authors:** Yuwen Zhang, Shouyue Sun, Ming Wang, Wenjuan Yu, Peizhan Chen, Fei Yuan, Xuqian Fang

**Affiliations:** ^1^ Department of Endocrine and Metabolic Diseases, Shanghai Institute of Endocrine and Metabolic Diseases, Ruijin Hospital, Shanghai Jiao Tong University School of Medicine, Shanghai, China; ^2^ Shanghai National Clinical Research Center for Metabolic Diseases, Key Laboratory for Endocrine and Metabolic Diseases of the National Health Commission of the PR China, Shanghai National Center for Translational Medicine, Ruijin Hospital, Shanghai, China; ^3^ Department of Neurosurgery, Ruijin Hospital, Shanghai Jiao Tong University School of Medicine, Shanghai, China; ^4^ Department of Ecology, Evolution, and Organismal Biology, Iowa State University, Ames, IA, United States; ^5^ Shanghai Institute of Digestive Surgery, Ruijin Hospital, Shanghai Jiao Tong University School of Medicine, Shanghai, China; ^6^ Department of Pathology, Ruijin Hospital, Shanghai Jiao Tong University School of Medicine, Shanghai, China

**Keywords:** hypopituitarism, untargeted metabolomics, creatine metabolism, beta-oxidation, pituitary hormones

## Abstract

**Objective:** Hypopituitarism (Hypo-Pit) is partial or complete insufficiency of anterior pituitary hormones. Besides hormone metabolism, the global metabolomics in Hypo-Pit are largely unknown. We aimed to explore potential biomarkers to aid in diagnosis and personalized treatment.

**Methods:** Using both univariate and multivariate statistical methods, we identified 72 differentially abundant features through liquid chromatography coupled to high-resolution mass spectrometry, obtained in 134 males with Hypo-Pit and 90 age matched healthy controls.

**Results:** Hypopituitarism exhibits an increased abundance of metabolites involved in amino acid degradation and glycerophospholipid synthesis, but decreased content of metabolites in steroid hormone synthesis and fatty acid beta-oxidation. Significantly changed metabolites included creatine, creatinine, L-alanine, phosphocholines, androstenedione, hydroprenenolone, and acylcarnitines. In Hypo-Pit patients, the increased ratio of creatine/creatinine suggested reduced creatine uptake and impaired creatine utilization, whereas the decreased level of beta-hydroxybutyrate, acetylcarnitine (C2) and a significantly decreased ratio of decanoylcarnitine (C10) to free carnitine suggested an impaired beta-oxidation. Furthermore, the creatine/creatinine and decanoylcarnitine/carnitine ratio were identified as diagnostic biomarkers for Hypo-Pit with AUCs of 0.976 and 0.988, respectively. Finally, we found that the creatinine and decanoylcarnitine/carnitine ratio could distinguish cases that were sensitive vs. resistant to human chorionic gonadotropin therapy.

**Conclusion:** We provided a global picture of altered metabolic pathways in Hypo-Pit, and the identified biomarkers in creatine metabolism and beta-oxidation might be useful for the preliminary screening and diagnosis of Hypo-Pit.

## Introduction

Hypopituitarism (Hypo-Pit) is a chronic endocrine illness with partial or complete insufficiency of the anterior pituitary caused by varied etiologies with a prevalence of 300–455 per million (Ascoli and Cavagnini, 2006), which has a high risk of cardiovascular morbidity and infertility (Stieg et al., 2017). Clinical manifestations of Hypo-Pit are variable and might be affected by the cause of hypopituitarism, age of onset, and the speed and degree of hormone secretion loss. Although a partial hormone deficiency that progresses slowly can go undetected for years, the sudden and complete loss of hormone secretion results in an emergency situation that requires immediate medical attention. Therefore, early diagnosis and prompt treatment is necessary.

The diagnosis of hypopituitarism is made by measuring basal hormone levels based on the morning fasting status or performing stimulation tests if necessary. Pituitary hormones can be diagnosed with basal hormone measurement. The diagnosis of growth hormone (GH) or adrenocorticotropic hormone (ACTH) deficiency requires provocation tests of the hypothalamic-anterior pituitary-target organ axis. However, there are several contraindications to these tests (Fleseriu et al., 2016). For example, an insulin tolerance test (ITT) is contraindicated in patients with ischemic heart disease, seizure disorders, or severe pituitary deficiency (Ghigo et al., 2008). Meanwhile, ITT has the potential risk of inducing severe hypoglycemia, convulsions, and coronary heart disease (Gómez et al., 2002), and there have even been reports of death in pediatric patients (Price, 1992). Easy and non-invasive diagnosis methods are needed as an alternative for special patients. As one of them, the determination of cortisol content in human scalp hair was recently used (Ibrahim and Van Uum, 2014). Metabolomics studies provide novel tools in identifying biomarkers of diseases early diagnosis and treatment efficacy (Taylor Fischer et al., 2019). Dooijeweert et al. found that untargeted metabolomics in dried blood spots is a useful tool for pyruvate kinase deficiency diagnosis (Van Dooijeweert et al., 2020). Souto-Carneiro et al. used serum metabolome and lipidome to identify potential biomarkers for rheumatoid arthritis and psoriatic arthritis (Souto-Carneiro et al., 2020). These suggested that evaluate the metabolic status of the Hypo-Pit patients may uncover the mechanisms of the diseases development and identify novel early diagnosis biomarkers and/or novel therapeutic targets.

Specific glucose and lipid profiles and biochemical changes have been identified in patients with growth hormone deficiency (López-Oliva et al., 2009; Sharma, 2018). Due to the lack of growth hormone, Hypo-Pit patients might suffer reduced lean body mass, increased body adiposity, reduced muscle strength, and abnormal lipid metabolism (Curtò and Trimarchi, 2016). Abnormal lipid metabolism might further lead to an increased prevalence of cardiovascular risk factors (Kearney et al., 2001; Cannavò et al., 2011). Gonadotrophin deficiency can induce spermatogenesis in men (Fang et al., 2019) and an increased risk of pregnancy complications for females (Du et al., 2014). GH has various regulatory influences on the metabolism of glucose and lipids via complex interactions with insulin and insulin-like growth factor-1 (Krsek, 2016). Mert et al. found dramatic reductions in oxygen consumption, carbon dioxide production, and energy expenditure in GH-releasing hormone (GHRH)−/− mice compared to those in wild-type mice, which might mediate the increased lifespan (Icyuz et al., 2020). Charlotte et al. discussed the possibility of serum metabolome in diagnosis of GH deficiency and for monitoring GH replacement. Although they found 13 metabolites useful in differentiating GH deficiency patients from controls, but large of them could not be structurally annotated (Höybye et al., 2014). Benefit from database assisted structure identification for metabolite identification, this problem can be solved to some extent (Menikarachchi et al., 2016). In this study, we aimed to identify metabolic changes in Hypo-Pit to explain its clinical phenotype and ultimately explore potential biomarkers to aid in diagnosis and personalized treatment.

## Materials and Methods

### Participant Recruitment

Patients and healthy controls were recruited at Ruijin Hospital (Shanghai, China) between Jan 2016 and Dec 2018. Eligible patients were clinically diagnosed with hypopituitarism based on clinical history, symptoms, biochemical parameters, and brain magnetic resonance imaging (MRI) tests. For congenital Hypo-Pit, patients were diagnosed with multiple pituitary hormone deficiency at a young age (∼6–12 years of age). The MRI scan suggested the presence of a transected or interrupted hypothalamic-pituitary stalk or pituitary hypoplasia. For acquired Hypo-Pit, patients were secondary to pituitary tumors, traumatic brain injury, pituitary irradiation, or a clear diction of dystocia history. For the healthy control group, participants were selected from local residents receiving annual physical examination, were free of acute or chronic diseases (including diabetes, cancer, and cardiovascular diseases), and had no symptoms of growth and developmental abnormalities. In order to alleviate the interference of gender in the serum metabolome (Dunn et al., 2015), and evaluate the effectiveness of hormone replacement therapy, we only selected young male patients in the current study. The discovery set included 135 Hypo-Pit patients and 90 age-matched male controls. The external validation set included another 122 male Hypo-Pit patients. The recruitment of the validation set followed the same protocols as described for the discovery set and was conducted between June 2018 and July 2019. The study protocol was approved by the Committee on Human Research at Ruijin Hospital, Shanghai Jiao Tong University School of Medicine, China. All patients provided informed written consent to participate in the study.

### Gonadotropin Treatment and Follow-Up

**FIGURE 1:**
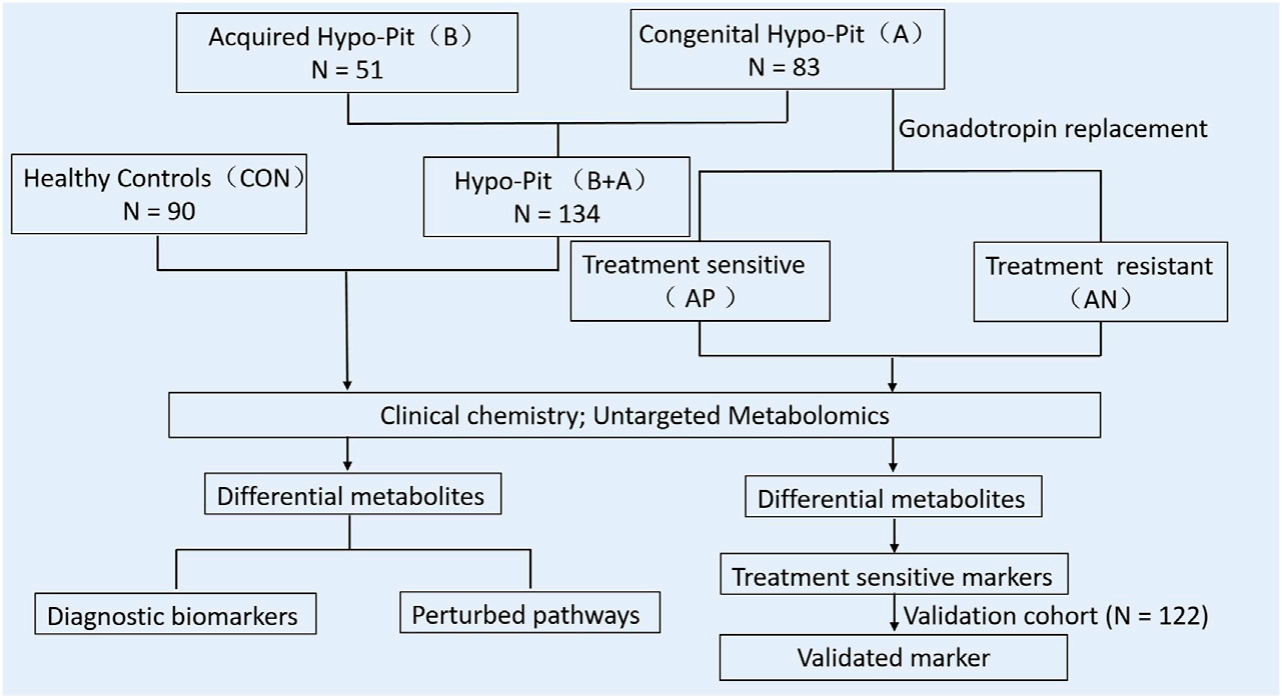
Flowchart of the study design in this work.

For Hypo-Pit, physiologic dosages of glucocorticoids and thyroid hormone were administered after diagnosis. Recombinant human GH was also recommended. Then, intramuscular human chorionic gonadotropin (hCG; 2000 IU, Livzon Pharmaceutical Co., Guangdong, China) was administered twice weekly for at least 24 months. Regular follow-ups were conducted at an interval of 3–6 months. Testicular size, serum gonadotrophins, testosterone, and sperm count were measured at each visit. Testicle volume was measured by two-dimension ultrasound. Patients with a mean testicle size ≥6 ml during gonadotrophin treatment at follow-up were considered sensitive to hCG therapy. Otherwise, patients were considered as resistant to the therapy. The experimental design of this study is summarized in Figure 1.

### Sample Collection and Preparation

Peripheral blood was collected in 7 ml blood collection vacuum tubes with separating gel and centrifuged for 15 min (1,500× g, 4°C) at fasting state. A serum sample aliquot (150 μL) was stored at −80°C until use. 100 μL of serum was transferred to EP tubes, mixed with 400 μL of methanol/acetonitrile (1:1, v/v), incubated for 10 min at −20°C, and then centrifuged at 14,000× *g* for 15 min at 4°C. The supernatants were dried with nitrogen and dissolved in 100 μL acetonitrile/water (1:1, v/v). To monitor the stability and repeatability of instrument analysis, quality control (QC) samples were prepared by pooling 30 μL of each sample. 13 QC samples were inserted every 18 real samples to monitor the ultra-performance liquid chromatography-quadrupole-time of flight-mass spectrometry (UPLC-Q-TOF-MS) response in real-time.

### UPLC-Q-TOF/MS Analysis

Metabolic profiling of serum samples was performed on an Agilent 1,290 Infinity LC system (Agilent Technologies, Santa-Clara, CA, United States) coupled with an AB SCIEX Triple TOF 6600 system (AB SCIEX, Framingham, MA, United States) (Zhou et al., 2019; Xu et al., 2021). Chromatographic separation was performed on an ACQUITY HSS T3 1.8 μm column (2.1 × 100 mm) for both positive and negative ion modes. The mobile phase was set as follows: A = 0.1% formic acid in water, B = 0.1% formic acid in acetonitrile, C = 0.5 mM ammonium fluoride in water and D = acetonitrile. In the positive (negative) mode, the elution gradient started with 1% B (D) for 1 min, linearly increased to 100% B (D) at 8 min, maintained for 2 min, and then returned to 1% B (D) for approximately 2 min of equilibrium. The delivery flow rate was 300 μL/min, and 2 μL aliquots of each sample were injected into the column. Electrospray ionization source conditions on Triple TOF were set as follows: ion source gas 1, 60 psi; ion source gas 2, 60 psi; curtain gas, 30 psi; source temperature, 600°C; and ionspray voltage floating, ±5500 V. Information-dependent acquisition, an artificial intelligence-based product ion scan mode, was used to detect and identify MS/MS spectra. The parameters were set as follows: collision energy, 35 V ± 15 eV; declustering potential, 60 V (+) and −60 V (−); exclude isotopes within 4 Da, candidate ions to monitor per cycle, 10. The LC-MS platform analysis was conducted with the assistance of Applied Protein Technology Co., Ltd. (Shanghai, China).

### Metabolomics Data Analysis

Raw UPLC-Q-TOF/MS data were converted to mzXML format using the Proteo Wizard MS converter tool and processed using XCMS online software. For peak picking, the following parameters were used: centwave m/z = 25 ppm, peakwidth = c (10, 60), prefilter = c (10, 100). For peak grouping, bw = 5, mzwid = 0.025, and minfrac = 0.5 were used. After normalization of total peak intensity, processed data were imported into SIMCA-P (version 14.1, Umetrics, Umea, Sweden) and subjected to multivariate data analysis, including Pareto-scaled principal component analysis (PCA) and orthogonal partial least-squares discriminant analysis (OPLS-DA). Seven-fold cross-validation and response permutation tests were used to evaluate the robustness of the model. The variable importance in the projection (VIP) value of each variable in the OPLS-DA model was calculated to indicate its contribution to class separation. Metabolites with VIP >1 were further analyzed with the univariate student’s t-test to measure the significance of each metabolite, and *p*-values < 0.05 were considered statistically significant. The relative expression of studied metabolites was used to perform hierarchical clustering analysis using Cluster 3.0 and Java Treeview software (http://jtreeview.sourceforge.net).

### Bioinformatics Analysis and Biomarker Identification

We compared the metabolic phenotypes of Hypo-Pit patients and healthy controls with a VIP value >1 and *p*-value < 0.05 to identify differential metabolites and to reveal perturbed metabolic pathways. KEGG (Kyoto Encyclopedia of Genes and Genomes) pathway enrichment analyses (www.metaboanalyst.ca) and the human metabolome database (https://hmdb.ca/) were used to perform biological function analyses based on Fisher’s exact test. Only pathways with *p*-values under 0.05 were considered significant. The same strategy was used to identify differential metabolites between congenital vs. acquired Hypo-Pit and between Hypo-Pit patients sensitive or resistant to hCG treatments. To identify potential diagnostic biomarkers, a receiver-operating characteristic curve (ROC) analysis was performed to quantify the diagnostic performance of individual metabolites. The area under the curve (AUC) was calculated with R software (version 4.0.3; www.r-project.org). Similar analyses of significant metabolites in gonadotropin replacement sensitive and gonadotropin replacement resistant congenital Hypo-Pit were performed to identify biomarkers of sensitivity to hormone substitute therapy.

### Statistical Analysis

Continuous variables were presented as the mean ± SD for normally distributed variables or median with interquartile range (IQR) for the skewed variables. The Kolmogorov-Smirnov statistical test was used to assess data normality. Student’s t-test, Mann-Whitney U-test and the Pearson chi-square test were used where appropriate. All analyses were performed with SPSS software version 23.0 (SPSS Inc., Chicago, IL, United States ). Significance tests were two tailed, and a *p* < 0.05 was considered statistically significant.

## Results

The discovery cohort included 83 individuals with congenital Hypo-Pit (mean age: 25.42 ± 5.79), 51 with acquired Hypo-Pit (mean age: 22.88 ± 6.04), and 90 heathy controls (mean age: 22.87 ± 2.13). In congenital Hypo-Pit, 25 patients were sensitive to gonadotropin treatment, whereas 58 patients were resistant. The clinical and laboratory characteristics and baseline comparison are summarized in Table 1. Beside weight, no significant difference was identified between the patients with Hypo-Pit and controls for age, height, and body mass index (BMI). Compared with the controls, Hypo-Pit patients had significantly lower levels of the hormones secreted by the pituitary gland (Table 1). The external validation set included another 122 male congenital Hypo-Pit patients (mean age: 25.75 ± 6.15), with 52 patients sensitive to gonadotropin treatment, whereas 70 patients were resistant. There is no significant difference between hCG-sensitive group and resistant group of GH deficiency, LH/FSH deficiency and the level of testosterone. In hCG-sensitive group, a decreased TSH and ACTH deficiency, and a relatively higher serum creatinine level was noticed (Table 2).

**TABLE 1:** Clinical characteristics of the participants in identification study population.

Characteristics^a^	Congenital hypopituitarism	Acquired hypopituitarism	Control group	*p*-value
	(*n* = 83)	(*n* = 51)	(*n* = 90)	—
Basic information				
Gender (male/female)	83/0	51/0	90/0	—
Age (year)	25.42 ± 5.79	22.88 ± 6.04	22.87 ± 2.13	0.061
Height (cm)	166.41 ± 8.51	169.61 ± 11.65	167.59 ± 5.47	0.102
Weight (kg)	65.88 ± 12.88	72.33 ± 19.50	66.20 ± 10.97	0.008
BMI (kg/m^2^)	23.69 ± 3.64	24.81 ± 4.74	23.53 ± 4.64	0.057
Pituitary hormone deficiency				
GH deficiency	100% (82/82)	98.0% (50/51)	—	—
LH/FSH deficiency	97.6% (80/82)	96.1% (49/51)	—	—
TSH deficiency	91.5% (75/82)	96.1% (49/51)	—	—
ACTH deficiency	90.2% (74/82)	94.1% (48/51)	—	—

a The data are the mean ± SD for continuous variables and *n* (%) for categorical variables. Abbreviations: BMI, body mass index; GH, growth hormone; LH, luteinizing hormone; FSH, follicle-stimulating hormone; TSH, thyrotropin; ACTH, adrenocorticotropic hormone.

**TABLE 2:** Clinical characteristics of the participants in validation cohort.

Characteristics^a^	Total	hCG-sensitive group	hCG-resistant group	*p*-value
	(*n* = 122)	(*n* = 52)	(*n* = 70)	—
Basic information				
Gender (male/female)	122/0	52/0	70/0	—
Age (year)	25.75 ± 6.15	22.88 ± 6.04	25.69 ± 6.79	0.883
Height (cm)	165.65 ± 8.35	165.90 ± 7.15	165.46 ± 9.19	0.767
Weight (kg)	65.88 ± 13.04	67.94 ± 12.89	64.34 ± 13.03	0.131
BMI	23.91 ± 3.79	24.58 ± 3.77	23.41 ± 3.74	0.090
Pituitary hormone deficiency				
GH deficiency	100% (122/122)	100% (52/52)	100% (70/70)	—
LH/FSH deficiency	98.4% (120/122)	98.1% (51/52)	98.6% (69/70)	0.673
TSH deficiency	89.3% (109/122)	82.7% (43/52)	94.3% (66/70)	0.040
ACTH deficiency	82.0% (100/122)	71.2% (37/52)	90.0% (63/70)	0.007
Serum creatinine (μmol/L)	69.7 ± 10.7	72.5 ± 10.9	67.7 ± 10.2	0.016
Testosterone (ng/ml)	<0.1 (<0.1–0.25)	<0.1 (<0.1- < 0.1)	<0.1 (<0.1–0.47)	—

a The data are the mean ± SD or median (quartile 1–3) for continuous variables and *n* (%) for categorical variables. Abbreviations: hCG, human chorionic gonadotropin; BMI, body mass index; GH, growth hormone; LH, luteinizing hormone; FSH, follicle-stimulating hormone; TSH, thyrotropin; ACTH, adrenocorticotropic hormone.

### Metabolic Characteristics of Hypopituitarism Patients

**FIGURE 2:**
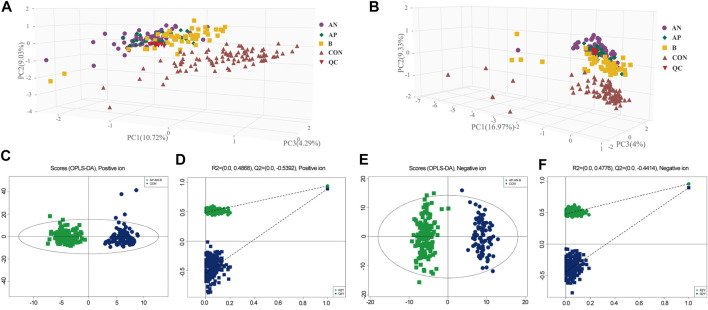
**(A,B).** PCA plot of hypopituitarism vs. healthy controls in positive ion and negative ion. AN, treatment sensitive of congenital Hypo-pit; AP, treatment resistant of congenital Hypo-pit, B: acquired Hypo-pit; CON, healthy controls; QC, quality control samples. Figures 2C,D **.** OPLS-DA plot of hypopituitarism vs. healthy controls in positive ion. Figures 2E,F **.** OPLS-DA plot of hypopituitarism vs. healthy controls in negative ion.

**FIGURE 3:**
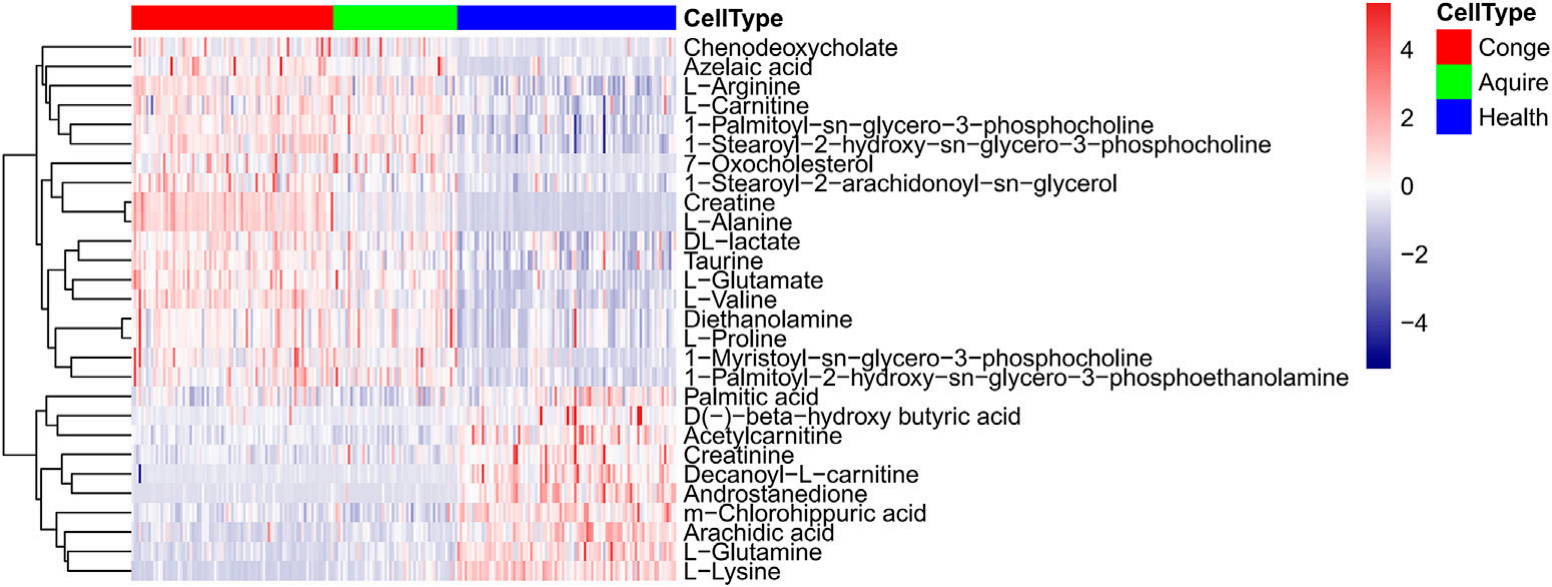
Heat plot of the top 28 differential metabolites in hypopituitarism vs. healthy controls.

In total 6,380 positive-mode and 6,577 negative-mode unique metabolite features were identified between 135 Hypo-Pit patients and 90 controls. After log transformation and scaling, QC samples clustered tightly in PCA score plots in both positive and negative modes with CV% of all metabolites (4.45%, range 1.1–44.07%), indicating satisfactory reproducibility (Figures 2A,B). The supervised orthogonal partial-least square discriminant analysis (OPLS-DA) plots identified distinct metabolic profiles between Hypo-Pit patients and healthy controls for both positive and negative modes (Figures 2C–F). In total, 72 annotated metabolites were identified with VIP >1 and *p* < 0.05 between Hypo-Pit and healthy control groups (Supplementary Table S1). Of them, 25 (34.7%) metabolites belong to the subclass of amino acids, 17 metabolites belong to fatty acids, 12 metabolites belong to glycerin phospholipid, four metabolites belong to steroids, three metabolites belong to nucleic acid, and two metabolites belong to bile acids and the others (Supplementary Figure S1). The heatmap for the top 28 differential metabolites was presented in Figure 3. These included the following: steroids: androstenedione, hydropregnenolone sulfate, pregnenolone sulfate, 7-oxocholesterol; amino acids: proline, alanine, valine, arginine, glutamate, creatinine, creatine and taurine; glycerophospholipids: diethanolamine, 1-palmitoyl-2-hydroxy-sn-glycero-3-phosphoethanolamine, 1-myristoyl-sn-glycero-3-phosphocholine, 1-palmitoyl-sn-glycero-3-phosphocholine, 1-stearoyl-2-hydroxy-sn-glycero-3-phosphocholine, 1-stearoyl-2-arachidonoyl-sn-glycerol; fatty acids: carnitine, decanoylcarnitine, acetylcarnitine, beta-hydroxybutyric acid, palmitic acid, azelaic acid, arachidic acid, and chlorohippuric acid.

**FIGURE 4:**
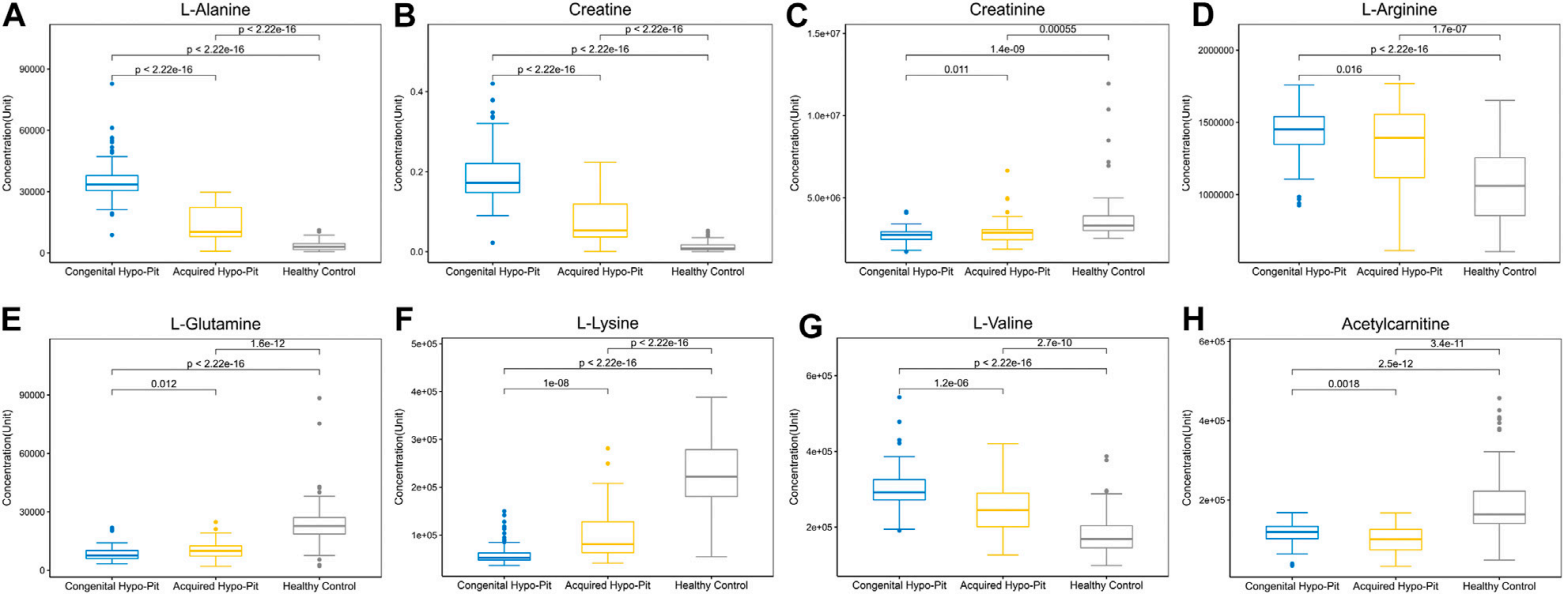
The expression pattern of typical differential metabolites.

OPLS-DA plots identified distinct metabolic profiles between congenital and acquired Hypo-Pit groups (Supplementary Figure S2). 57 annotated metabolites were identified with VIP >1 and *p* < 0.05 **(** Supplementary Table S2 **)**. There are 32 metabolites shows significant difference (*p* < 0.05) both in the comparison of Hypo-Pit vs. healthy controls and comparison of congenital Hypo-Pit vs. acquired Hypo-Pit patients (Table 3). The expression pattern of typical differential metabolites including L-alanine, creatine, creatinine, L-arginine, L-glutamine, L-lysine, L-valine and acetylcarnitine was shown in Figure 4.

**TABLE 3:** Differential metabolites shared in comparisons of comparison A (Hypo-Pit vs. healthy controls), comparison B (congenital vs. acquired Hypo-Pit), and comparison C (hCG-sensitive vs. hCG-resistant).

Name	Metabolites^a^	(AN + AP + B) vs. HCs^b^			(AN + AP) vs. B^b^			AP vs. AN^b^		
		VIP	Fold	*P*	VIP	Fold	*P*	VIP	Fold	*P*
M114T170	Creatinine	9.68	0.74	0.00	7.37	0.90	0.01	6.36	1.07	0.04
M132T350_2	Creatine	5.95	9.28	0.00	8.20	2.46	0.00	—	—	—
M124T294_2	Taurine	4.55	1.24	0.00	4.13	1.17	0.00	—	—	—
M175T505_2	L-Arginine	4.30	1.29	0.00	1.71	1.07	0.02	1.31	0.92	0.01
M188T502	L-Lysine	4.15	0.34	0.00	2.98	0.59	0.00	—	—	—
M116T298	L-Valine	4.04	1.57	0.00	3.13	1.22	0.00	—	—	—
M164T255_2	L-Phenylalanine	3.60	1.43	0.00	2.86	1.20	0.00	3.02	0.89	0.04
M203T256	L-Tryptophan	2.18	1.52	0.00	2.20	1.27	0.00	—	—	—
M128T300_2	L-Pyroglutamic acid	4.02	1.28	0.00	1.28	0.83	0.04	—	—	—
M156T433	L-Histidine	1.61	1.49	0.00	1.43	1.25	0.01	—	—	—
M90T350	L-Alanine	1.57	7.97	0.00	2.27	2.51	0.00	—	—	—
M118T303	Betaine	1.48	1.38	0.00	2.18	1.32	0.00	—	—	—
M130T309	L-Leucine	1.01	1.15	0.00	3.30	1.20	0.01	1.17	0.88	0.04
M391T151	Chenodeoxycholate	2.74	2.54	0.00	1.60	1.25	0.01	—	—	—
M165T154	Dihydrothymine	1.99	0.76	0.00	1.10	1.13	0.00	—	—	—
M496T191_3	1-Palmitoyl-sn-glycero-3-phosphocholine	28.91	1.31	0.00	—	—	—	23.27	0.93	0.01
M524T187_3	1-Stearoyl-2-hydroxy-sn-glycero-3-phosphocholine	20.89	1.35	0.00	5.77	1.06	0.03	—	—	—
M468T195	1-Myristoyl-sn-glycero-3-phosphocholine	5.03	2.66	0.00	—	—	—	7.12	0.59	0.00
M628T193	1-Stearoyl-2-arachidonoyl-sn-glycerol	2.87	1.61	0.00	3.18	1.36	0.00	—	—	—
M141T342_2	2-Oxoadipic acid	16.87	1.09	0.00	10.37	1.05	0.00	—	—	—
M162T355_2	L-Carnitine	5.56	1.12	0.00	—	—	—	4.70	0.92	0.01
M89T100	DL-lactate	6.87	1.33	0.00	1.00	1.25	0.01	—	—	—
M277T48	All *cis*-(6,9,12)-Linolenic acid	2.46	1.22	0.01	3.29	1.29	0.01	—	—	—
M120T260_2	Tyramine	2.16	1.14	0.01	—	—	—	1.93	0.88	0.01
M169T56	3-Hydroxycapric acid	1.62	0.63	0.00	1.27	1.23	0.00	—	—	—
M281T101_2	Oleic acid	1.49	1.10	0.03	1.02	1.12	0.04	—	—	—
M165T79	3-(2-Hydroxyphenyl)propionic acid	1.42	4.18	0.00	2.49	3.21	0.03	—	—	—
M137T290	1-Methylnicotinamide	1.05	2.38	0.00	1.10	1.55	0.02	—	—	—
M188T197	DL-Indole-3-lactic acid	1.43	2.50	0.00	1.57	1.81	0.00	—	—	—
M70T315	Diethanolamine	1.39	1.43	0.00	—	—	—	1.12	0.87	0.05
M149T119	D-Lyxose	1.27	0.73	0.00	2.68	0.81	0.03	—	—	—

a Differential metabolites selection criteria: VIP >1 and *p* < 0.05.

b A, congenital hypopituitarism; B, acquired hypopituitarism; AP, hCG-sensitive group with congenital hypopituitarism; AN, hCG-resistant group with congenital hypopituitarism; HC, health controls.

### Metabolic Perturbed Pathways in Hypopituitarism Patients

**FIGURE 5:**
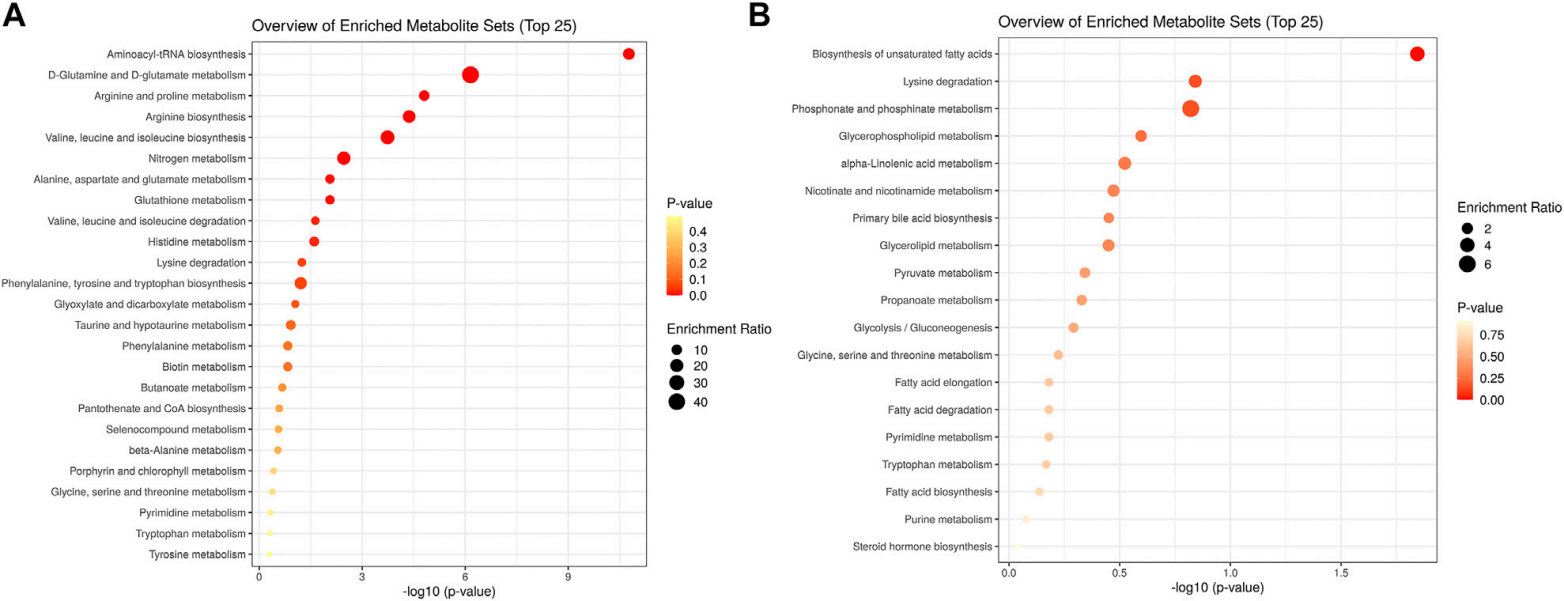
KEGG pathway enrichment analyses of the differential metabolites in hypopituitarism vs. healthy controls **(A)**. enrichment of differential metabolites of amino acids; **(B)**. enrichment of differential metabolites of non-amino acid metabolites.

Using KEGG pathway enrichment analyses by metaboanalyst software, the 25 differential amino acids clustered to pathways of aminoacl-tRNA biosynthesis, D-glutamine and D-glutamate metabolism, valine, leucine and glutamate metabolism, nitrogen metabolism, and arginine and proline metabolism (Figure 5A **)**. The 48 non-amino acid metabolites clustered to pathways of phosphonate and phosphonate metabolism, biosynthesis of unsaturated fatty acids, lysine degradation and glycerophospholipid metabolism (Figure 5B **)**. In arginine and proline metabolism, significant differential metabolites included creatine (VIP = 5.94, FC = 9.28), creatinine (VIP = 9.68, FC = 0.74), and L-arginine (VIP = 4.30, FC = 1.29). In valine, leucine, and isoleucine degradation pathways, significant differential metabolites included L-alanine (VIP = 1.57, FC = 7.96) and ketoisocaproic acid (VIP = 3.92, FC = 0.76). In unsaturated fatty acid metabolism, significant differential metabolites included carnitine (VIP = 5.56, FC = 1.12), decanoylcarnitine (VIP = 4.90, FC = 0.15), and acetylcarnitine (VIP = 2.67, FC = 0.57). In glycerophospholipid metabolism, significant differential metabolites included 1-palmitoyl-sn-glycero-3-phosphocholine (VIP = 28.91, FC = 1.31) and 1-stearoyl-2-hydroxy-sn-glycero-3-phosphocholine (VIP = 20.89, FC = 1.35). In steroid hormone biosynthesis, significant differential metabolites included androstenedione (VIP = 2.48, FC = 0.08) and hydropregnenolone sulfate (VIP = 2.45, FC = 0.21).

### Biomarkers for Diagnostics Hypo-Pit

**FIGURE 6:**
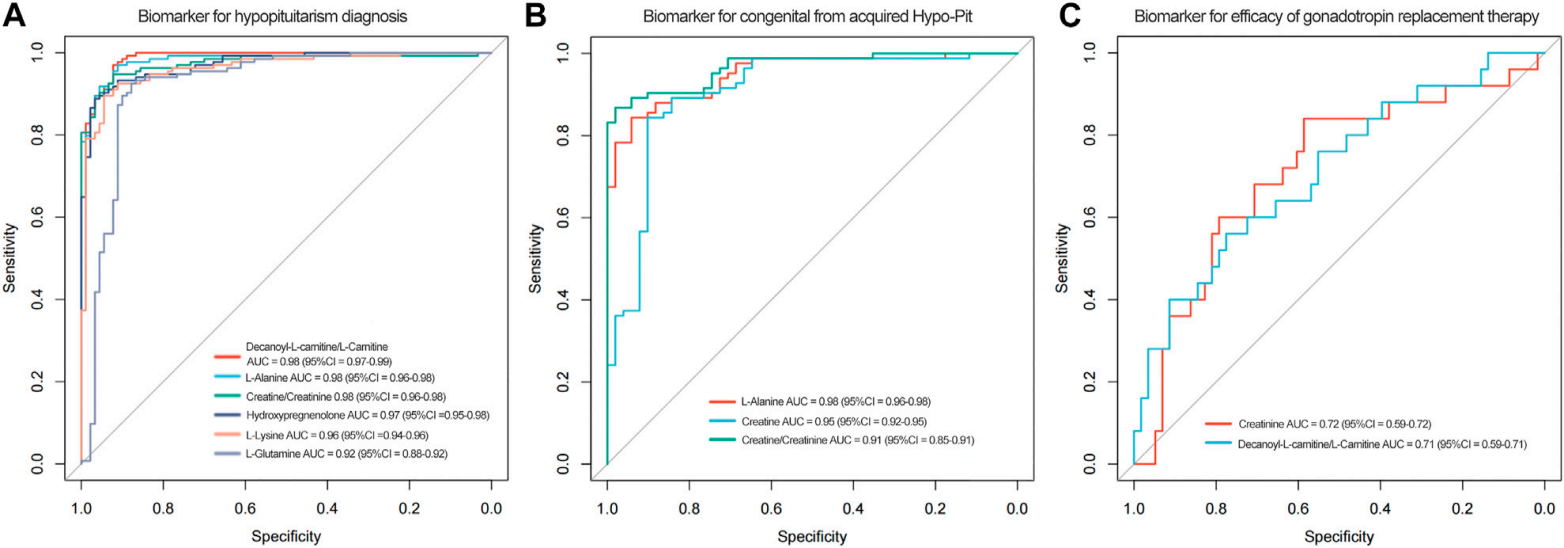
Figure 6A. The ROC curves of decanoylcarnitine/L-carnitine ratio, L-alanine, creatine/creatinine ratio, hydroprenenolone sulfate, L-lysine and L-glutamine for hypopituitarism diagnosis. Figure 6B **.** ROC curves of serum L-alanine, creatine and creatinine for discrimination of congenital from acquired Hypo-Pit patients. Figure 6C **.** ROC curves of creatinine and decanoylcarnitine/L-carnitine for efficacy of gonadotropin replacement therapy.

To identify potential biomarkers for Hypo-Pit diagnosis, ROC analyses were performed to quantify the diagnostic performance of metabolites. Sixteen metabolites with AUCs greater than 0.8 are shown in Table 4. The L-alanine, decanoyl-L-carnitine, creatine, hydropregnenolone-sulfate, L-lysine, androstanedione, L-glutamine, arachidic-acid, L-glutamate and L-valine were identified as potential diagnostic biomarkers for Hypo-Pit with AUC >0.9. Interestingly, the diagnostic performance of the creatine/creatinine ratio was better than that of creatine or creatinine alone, with an AUC of 0.976 (95% CI 0.957–0.994) compared to 0.894 for creatine and 0.857 for creatinine, respectively (*p* < 0.05). A similar pattern was observed for the decanoylcarnitine/carnitine ratio, with an AUC of 0.988 compared to that of 0.981 for decanoylcarnitine and 0.822 for carnitine **(** Figure 6A and Table 4 **)**. For the discrimination of congenital out of acquired Hypo-Pit, metabolites of L-alanine, creatine, L-lysine, L-valine, and creatine/creatinine were identified as biomarker with well accuracy (AUC >0.9; Figure 6B and Supplementary Table S3).

**TABLE 4:** Biomarkers for diagnostic of hypopituitarism and biomarkers for prediction of gonadotropin replacement therapy.

Metabolites	(A + B) vs. HCs^a^						AP vs. AN^a^					
	AUC (95%CI)	Threshold	Sensitivity	Specificity	PPV	NPV	AUC (95%CI)	Threshold	Sensitivity	Specificity	PPV	NPV
Creatine	0.89 (0.85–0.94)	94,506	0.82	0.93	0.89	0.87	—	—	—	—	—	—
Creatinine	0.86 (0.81–0.86)	2,947,373	0.75	0.88	0.70	0.90	0.72 (0.59–0.72)	2,687,014	0.84	0.59	0.47	0.89
L-Arginine	0.82 (0.77–0.88)	1,256,933	0.77	0.76	0.69	0.82	0.69 (0.56–0.69)	1,349,494	0.56	0.84	0.60	0.82
Creatine/Creatinine	0.98 (0.96–0.98)	0.03	0.95	0.92	0.94	0.92	—	—	—	—	—	—
L-Alanine	0.98 (0.96–0.98)	6,305	0.97	0.91	0.94	0.95	—	—	—	—	—	—
L-Valine	0.89 (0.85–0.94)	239,591	0.77	0.90	0.72	0.92	—	—	—	—	—	—
L-Lysine	0.96 (0.94–0.96)	60,229	0.89	0.94	0.96	0.85	—	—	—	—	—	—
L-Glutamate	0.90 (0.86–0.90)	60,398	0.87	0.84	0.89	0.81	—	—	—	—	—	—
L-Glutamine	0.92 (0.88–0.92)	14,157	0.93	0.88	0.92	0.88	—	—	—	—	—	—
Arachidic-acid	0.91 (0.87–0.91)	25,893	0.82	0.85	0.89	0.76	—	—	—	—	—	—
Androstanedione	0.96 (0.93–0.98)	20,059	0.94	0.90	0.93	0.91	—	—	—	—	—	—
Hydroxypregnenolone	0.97 (0.95–0.98)	31,817	0.93	0.90	0.94	0.92	—	—	—	—	—	—
L-Carnitine	0.82 (0.76–0.88)	6,775,600	0.73	0.88	0.68	0.89	—	—	—	—	—	—
Decanoyl-L-carnitine	0.98 (0.96–0.99)	90,526	0.96	0.92	0.95	0.93	0.67 (0.54–0.67)	6,966,037	0.64	0.70	0.48	0.82
Acetylcarnitine	0.85 (0.79–0.91)	139,630	0.87	0.77	0.85	0.80	—	—	—	—	—	—
Decanoyl-L-carnitine	0.98 (0.97–0.99)	0.01372	0.97	0.92	0.95	0.90	0.71 (0.59–0.71)	0.00783	0.56	0.78	0.52	0.80
/L-Carnitine												

a A, congenital hypopituitarism; B, acquired hypopituitarism; AP, hCG-sensitive group with congenital hypopituitarism; AN, hCG-resistant group with congenital hypopituitarism; HC, health controls. Abbreviations: ROC, receiver operator characteristic curve; PPV, positive predictive value; NPV, negative predictive value.

### Metabolic Profiles Reflect Sensitivity to Gonadotropin Replacement

To explore metabolite profiles in relation to clinical phenotype, OPLS-DA were performed to distinguished hCG-sensitive and hCG-resistant groups **(** Supplementary Figure S3 **)**. A total of 12 differential metabolites were identified with VIP >1 and *p* < 0.05 (Supplementary Table S4 **)**. According to the ROC analyses, creatinine and decanoylcarnitine/L-carnitine ratios were shown to be moderate predictors of gonadotropin replacement in discriminating hCG-sensitive and hCG-resistant patients with AUCs of 0.72, and 0.71, respectively (Table 4; Figure 6C). Creatinine usually detected by alkaline picrate method in medical services. Retrospectively analysis shows serum creatinine has a better AUC of 0.75. In further, creatinine was evaluated in an external validation cohort including hCG-sensitive (n = 52) and hCG-resistant (*n* = 70) patients. Patients in the hCG-sensitive group had higher levels of serum creatinine than those in the resistant group (72.5 ± 10.9 vs. 67.7 ± 10.2, *p* < 0.001, Table 2) with an AUC of 0.716 (95% CI: 0.608–0.825).

## Discussion

### General Aspects

The study revealed the metabolic profiles of patients with Hypo-Pit and identified potential biomarkers for diagnosing or predicting the outcomes of gonadotropin therapy. Diagnosis of Hypo-Pit in the elderly or patients with severe heart disease or seizures is challenging, as the standard hormone provocation tests hold higher risks. Here, we found that amino acids, unsaturated fatty acids, glycerophospholipids and steroid hormone biosynthesis were significantly altered and closely associated with the clinical phenotype. These might serve as potential biomarkers for the early detection of Hypo-Pit, especially in patients who cannot tolerate the standard hormone provocation test. Importantly, the metabolites, especially creatinine could also be used in the early prediction of hCG therapy efficacy, which could guide clinicians to use increased dosages of hCG in advance for the patients who are considered resistant.

### Increased Degradation of Amino Acids

**FIGURE 7:**
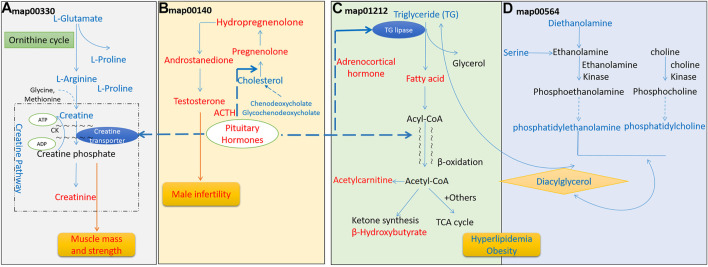
Perturbed metabolism signaling pathways in hypopituitarism. **(A)**. Perturbed arginine and proline metabolism; **(B)**. Perturbed steroid hormone synthesis; **(C)**. Perturbed fatty acid metabolism; **(D)**. Perturbed glycerophospholipid synthesis. Metabolites in blue increased in the serum, and metabolites in red decreased.

In 25 differential amino acids metabolites, a large proposition (17/25) is significantly increased in Hypo-Pit, which suggested an increased degradation of amino acids, similar to finding of growth hormone receptor deficient pig model (Riedel et al., 2020). In the arginine and proline metabolism pathway **(** Figure 7A **)**, creatine and upstream precursor substances (glutamate, proline, and arginine) significantly increased. In contrast, creatinine, which is converted by non-enzymatic dehydration and cyclization from creatine and phosphocreatine, significantly decreased. Creatine phosphate provides energy of motion to promote muscle mass and strength (Jowko et al., 2001). Consistent with this, both muscle mass and strength decreased in the Hypo-Pit group **(** Table 5 **)**. As verification, serum creatinine detected by the alkaline picrate method was lower in the Hypo-pit cohort (70.1 ± 9.6 μmol/L vs. 88.5 ± 26.5 μmol/L, *p* < 0.001), which is consistent with results detected by the untargeted metabolomics method. Creatine is physiologically provided in the diet via by endogenous synthesis from arginine, glycine, and methionine in the kidneys and liver. In our study, the creatine concentration was significantly increased, but creatinine was decreased in the peripheral blood. With no evidence of creatine supplementation from diet, a creatine utilization disorder in Hypo-Pit is likely. Creatine is mainly used in muscle and brain and is regulated through SLC6A8-mediated active uptake (Colas et al., 2020). The mechanisms mediate the functions of SLC6A8 are not fully understood. Previous studies showed that SLC6A8 can be modulated by substrate availability (Zervou et al., 2013), kinases and phosphatases (Kristensen et al., 2011), and the modulation of mature protein function (Brown et al., 2014; Ndika et al., 2014). The role of sex hormones is currently hypothetical, which might explain unexpected sex-based differences in metabolic patterns of urinary excretion of creatine and guanidinoacetate (Joncquel-Chevalier Curt et al., 2013). In a hypophysectomized rat model, creatine content in muscle was greatly decreased and normalized after GH administration, indicating that GHs might have a role in controlling creatine uptake by muscle (Tan and Ungar, 1979). Thyroid hormone treatment resulted in an approximately 50% reduction in intracellular creatine and creatine phosphate and a reduction in SLC6A8 mRNA (Queiroz et al., 2002). In addition, insulin and insulin-like compounds have been reported to have a direct stimulatory effect on creatine uptake by muscle (Tomcik et al., 2017). Therefore, creatine uptake might be regulated by pituitary-secreted hormones.

**TABLE 5:** Anthropometric and biochemical characteristics of the study population.

Characteristics	Hypopituitarism group	Control group	*p*-value
	(*n* = 134)	(*n* = 90)	—
Muscle Mass			
Soft lean mass	42.79 ± 8.87	47.69 ± 7.42	0.005
Skeletal muscle mass	26.70 ± 5.62	32.15 ± 4.68	0.004
Muscle strength			
Handgrip strength	33.62 ± 10.65	47.65 ± 2.75	<0.001
Serum creatinine (μmol/L)	70.1 ± 9.6	88.5 ± 26.5	<0.001
Lipid profile (mmol/L)			
Triglycerides	2.42 ± 2.98	1.13 ± 0.57	<0.001
Total cholesterol	5.05 ± 1.27	4.02 ± 1.69	<0.001

### Decreased Steroid Hormone Biosynthesis

In the steroid hormone biosynthesis pathway (Figure 7B), the synthetic precursors of steroid cholesterol (7-oxocholesterol) and chenodeoxycholate increased, but steroid hormones and metabolites, including hydropregnenolone sulfate, pregnenolone sulfate, and androstenedione, were remarkably decreased. In clinical findings, Hypo-Pit patients had significantly higher total cholesterol, detected by the enzymatic method (5.05 ± 1.27 mmol/L vs. 4.02 ± 1.69 mmol/L, *p* < 0.001), and obvious testosterone deficiencies (Table 5). It was reasonable that precursors or metabolites of steroid hormones were remarkably decreased, which is similar to the finding that men with Hypo-Pit have very severe overall steroid hormone deficiency (Giton et al., 2015). The steroid hormone deficiency also supports the credibility of experimental data detected by untargeted metabolomics.

### Decreased Fatty Acid β-oxidation

For the fatty acid metabolism pathway (Figure 7C), decreased level of β-hydroxybutyrate, acylcarnitine (C2) and a significantly decreased ratio of decanoylcarnitine (C10) to free carnitine suggested an impaired β-oxidation. The ketone body β-hydroxybutyrate was produced from the incomplete β-oxidation of fatty acids in the liver, to provides an essential carrier of energy from the liver to peripheral tissues (Newman and Verdin, 2017). Acylcarnitine (C2) is the end-products of different long-chain fatty acid oxidation in mitochondria. The decreased level of acylcarnitine (C2), and accumulations of disease-specific acylcarnitines due to blockage in the carnitine cycle were frequently observed in fatty acid β-oxidation disorder (Law et al., 2007). Abnormal carnitine metabolism is related to fatty acid oxidation disorder (Rattray et al., 2019). Reduced β-oxidation was observed in the Hypo-pit group with the clinical phenotype of hyperlipidemia and increased triglycerides detected by the enzymatic method (Table 5). Our data show that Hypo-Pit is a type of hyperlipidemia with lipid metabolism disorder, in which the levels of 3-hydroxycapric acid, palmitic acid, arachidic acid, azelaic acid, and β-hydroxybutyrate are decreased. These fatty acids are substrates or by-products in β fatty acid oxidation pathways (Moczulski et al., 2009). We suspected at least two obstacles in triglyceride (TG) utilization. One was in adipose lipid mobilization; its rate-limiting enzyme hormone-sensitive triglyceride lipase (HSTL) is a hormone-sensitive lipase regulated by hormones, such as ACTH (Dichek et al., 2006). The other was in the carnitine shuttle system and mitochondrion β-oxidation. The movement of long-chain fatty acid into mitochondrion depending enzyme carnitine palmityl transferase I (CPT-I) activity (Bremer and Norum, 1967). CPT-I can be regulated by hormone stimulation, such as by insulin (Dobbins et al., 2001; Obici et al., 2003), glucagon (Brady and Brady, 1989), and thyroid hormones (Zhang et al., 2004). Carnitine and acetylcarnitine are important in the acquisition and maintenance of sperm motility. Combining carnitine and acetylcarnitine with micronutrients has been investigated as a treatment for infertility in men (Li et al., 2005). Recently, a double blind, randomized, placebo-controlled trial on the effect of carnitine and acetylcarnitine on sperm parameters in men with idiopathic oligoasthenozoospermia showed that combined treatment was beneficial for male infertility (Micic et al., 2019). Gonadotrophin deficiency and male infertility is a common symptom in young men with Hypo-Pit (Rottembourg et al., 2008). It is important to evaluate whether Hypo-Pit can benefit from combined acetylcarnitine treatment to improve fertility.

### Increased Glycerophospholipid Biosynthesis

In glycerophospholipid metabolism (Figure 7D), serine, diethanolamine, phosphatidylethanolamine, phosphatidylcholine, and diacylglycerol are substrates or by-products in the glycerophospholipid pathway. They were increased in the Hypo-Pit group as compared to levels in healthy controls. The increased glycerophospholipid biosynthesis in Hypo-Pit may be due to increased level of triglyceride.

### Biomarkers for Diagnostics Hypo-Pit and Gonadotropin Replacement Sensitivity

In clinical applications, decanoylcarnitine/L-carnitine ratio, L-alanine, and creatine/creatinine ratio reached promising diagnostic accuracy (AUC >0.95) in hypopituitarism diagnosis. Of them, creatinine and decanoylcarnitine/L-carnitine also find effective to predict the outcomes of gonadotropin replacement therapy. Interestingly, these biomarkers have relationship with mitochondrial function. Lactate and L-alanine are widely used clinically as biomarkers of mitochondrial dysfunction (McMillan et al., 2014). Mitochondrion is a key organelle in cellular bioenergetics. Mitochondrial dysfunction can not only rooted form inherited disorders, but also secondary MD-related diseases (e.g., type 2 diabetes, obesity and neurodegenerative diseases) (Ostojic, 2017). As growth hormone (GH) and the insulin-like growth factor-1 (IGF-1) has function on mitochondrial biogenesis, respiration and ATP production, oxidative stress, senescence, and apoptosis (Poudel et al., 2020). Thus, it was possible that hypo-pit patients may have secondary MD-related diseases. Creatine is a universal energy currency for cell metabolism, which is partly synthesized in mitochondria. When mitochondria become dysfunctional, creatine synthesis or utilization might be disturbed, with creatine perhaps discharged to the blood (Barbieri et al., 2016). In respiratory chain disease, creatine found to be reproducibly elevated in two independent cohorts, exceeding lactate and alanine in magnitude of elevation and statistical significance (Shaham et al., 2010).

Several limitations should be acknowledged in this study. First, creatine and creatinine were determined in the serum of the patients, but intracellular creatine and creatine phosphate in muscles could not be tested in patients due to ethical considerations. Second, our study focused on the efficacy of hCG therapy, instead of human menopausal gonadotropin because of economic factors. Third, β-oxidation was not clearly described as acylcarnitine profiling assay was not present available for us.

In summary, increased degradation of amino acids and glycerophospholipid biosynthesis, decreased steroid hormone biosynthesis and fatty acid β-oxidation were identified by untargeted metabolism. These perturb pathways support the pathophysiology of Hypo-Pit with respect to decreased muscle strength and content, male infertility, hyperlipidemia, and obesity. The increased creatine and L-alanine in serum may indicate mitochondrial dysfunction in Hypo-pit. Moreover, the identified biomarkers in creatine metabolism and β-oxidation might be useful for preliminary screening and predicting the sensitivity of gonadotropin hormone substitution therapy for Hypo-Pit.

## Data Availability

The raw data supporting the conclusion of this article will be made available by the authors, without undue reservation.
