# Supplementary material for: Untargeted LC/MS-Based Metabolic Phenotyping of Hypopituitarism in Young Males
Source: Front Pharmacol. 2021 Jul 8;12:684869. doi: 10.3389/fphar.2021.684869 (PMC8295757; doi:10.3389/fphar.2021.684869)
Supplement: Supplementary file 1 [file Table1.docx]

**Supplement Table 1.** Differential metabolites between hypo-pit and controls

| **Name** | **Adduct** | **Description** | **VIP** | **Fold** | ***P*-value** | **m/z** | **rt(s)** |
| --- | --- | --- | --- | --- | --- | --- | --- |
| M125T92 | M+ | 3-Methylhistamine | 1.23 | 0.72 | 0.00 | 125.09 | 91.68 |
| M118T303 | (M+H)+ | Betaine | 1.48 | 1.38 | 0.00 | 118.09 | 303.04 |
| M132T350_2 | (M+H)+ | Creatine | 5.95 | 9.28 | 0.00 | 132.08 | 350.02 |
| M114T170 | (M+H)+ | Creatinine | 9.68 | 0.74 | 0.00 | 114.07 | 170.48 |
| M188T197 | (M+H-H2O)+ | DL-Indole-3-lactic acid | 1.43 | 2.50 | 0.00 | 188.07 | 196.58 |
| M89T227_2 | (M-H)- | DL-lactate | 6.87 | 1.33 | 0.00 | 89.02 | 226.56 |
| M149T119 | (M-H)- | D-Lyxose | 1.27 | 0.73 | 0.00 | 149.04 | 119.21 |
| M157T298 | (M+CH3CN+H)+ | D-Proline | 1.12 | 0.42 | 0.00 | 157.10 | 297.93 |
| M90T350 | (M+H)+ | L-Alanine | 1.57 | 7.97 | 0.00 | 90.05 | 350.03 |
| M175T505_2 | (M+H)+ | L-Arginine | 4.30 | 1.29 | 0.00 | 175.12 | 505.38 |
| M162T355_2 | (M+H)+ | L-Carnitine | 5.56 | 1.12 | 0.00 | 162.11 | 354.84 |
| M146T399 | (M-H)- | L-Glutamate | 2.12 | 1.96 | 0.00 | 146.05 | 398.86 |
| M188T352 | (M+CH3CN+H)+ | L-Glutamine | 1.13 | 0.39 | 0.00 | 188.10 | 352.01 |
| M154T399 | (M-H)- | L-Histidine | 1.52 | 1.39 | 0.00 | 154.06 | 399.41 |
| M130T309 | (M-H)- | L-Leucine | 1.01 | 1.15 | 0.00 | 130.09 | 309.14 |
| M188T502 | (M+CH3CN+H)+ | L-Lysine | 4.15 | 0.34 | 0.00 | 188.14 | 502.13 |
| M173T245 | (M+CH3CN+H)+ | L-Norleucine | 1.31 | 0.33 | 0.00 | 173.13 | 244.54 |
| M164T255_2 | (M-H)- | L-Phenylalanine | 3.60 | 1.43 | 0.00 | 164.07 | 255.32 |
| M116T315 | (M+H)+ | L-Proline | 1.63 | 1.47 | 0.00 | 116.07 | 314.73 |
| M128T300_2 | (M-H)- | L-Pyroglutamic acid | 4.02 | 1.28 | 0.00 | 128.04 | 300.39 |
| M203T256 | (M-H)- | L-Tryptophan | 2.18 | 1.52 | 0.00 | 203.08 | 256.45 |
| M116T298 | (M-H)- | L-Valine | 4.04 | 1.57 | 0.00 | 116.07 | 298.45 |
| M213T168 | M- | m-Chlorohippuric acid | 1.20 | 0.80 | 0.00 | 213.02 | 168.42 |
| M203T483 | (M+H)+ | NG,NG-dimethyl-L-arginine(ADMA) | 1.34 | 1.20 | 0.02 | 203.15 | 483.27 |
| M174T496 | (M+CH3CN+H)+ | Ornithine | 1.79 | 0.38 | 0.00 | 174.12 | 496.20 |
| M124T294_2 | (M-H)- | Taurine | 4.55 | 1.24 | 0.00 | 124.01 | 294.33 |
| M212T32 | (M-H)- | Indoxyl sulfate | 6.10 | 1.28 | 0.02 | 212.00 | 31.96 |
| M391T151 | (M-H)- | Chenodeoxycholate | 2.74 | 2.54 | 0.00 | 391.28 | 151.28 |
| M448T210 | (M-H)- | Glycochenodeoxycholate | 1.41 | 1.46 | 0.03 | 448.31 | 209.55 |
| M160T385 | (M+CH3COO+2H)+ | Cyclohexylamine | 1.49 | 1.28 | 0.01 | 160.13 | 384.97 |
| M319T54 | (M-H)- | (+-)12-HETE | 1.88 | 2.29 | 0.00 | 319.23 | 53.73 |
| M146T378_2 | M+ | (3-Carboxypropyl)trimethylammonium cation | 2.15 | 0.83 | 0.00 | 146.12 | 377.79 |
| M319T36 | (M-H)- | 12(R)-HETE | 1.01 | 2.03 | 0.00 | 319.23 | 36.38 |
| M295T49 | (M-H)- | 13(S)-HODE | 1.17 | 1.26 | 0.00 | 295.23 | 48.71 |
| M313T35_3 | (M+H-H2O)+ | 1-Palmitoylglycerol | 1.06 | 1.16 | 0.00 | 313.27 | 34.56 |
| M117T162 | (M-H)- | 2-Hydroxy-3-methylbutyric acid | 1.38 | 1.22 | 0.01 | 117.06 | 162.20 |
| M204T308 | (M+H)+ | Acetylcarnitine | 2.67 | 0.57 | 0.00 | 204.12 | 307.88 |
| M277T48 | (M-H)- | all cis-(6,9,12)-Linolenic acid | 2.46 | 1.22 | 0.01 | 277.22 | 47.64 |
| M311T36 | (M-H)- | Arachidic acid | 1.32 | 0.54 | 0.00 | 311.29 | 36.22 |
| M187T346 | (M-H)- | Azelaic acid | 3.07 | 2.61 | 0.00 | 187.10 | 345.86 |
| M103T235 | (M-H)- | D(-)-beta-hydroxy butyric acid | 1.37 | 0.38 | 0.00 | 103.04 | 235.12 |
| M316T188 | M+ | Decanoyl-L-carnitine | 4.90 | 0.15 | 0.00 | 316.25 | 187.87 |
| M337T32 | (M+H-H2O)+ | MG(18:2(9Z,12Z)/0:0/0:0)[rac] | 1.52 | 1.70 | 0.00 | 337.27 | 31.84 |
| M209T35 | (M+H-H2O)+ | Myristoleic acid | 1.23 | 2.84 | 0.00 | 209.19 | 34.53 |
| M281T101_2 | (M-H)- | Oleic acid | 1.49 | 1.10 | 0.03 | 281.25 | 101.04 |
| M255T48 | (M-H)- | Palmitic acid | 7.28 | 0.85 | 0.00 | 255.23 | 47.77 |
| M201T329 | (M-H)- | Sebacic acid | 1.39 | 1.47 | 0.01 | 201.11 | 329.38 |
| M809T90 | (M+Na)+ | 1,2-dioleoyl-sn-glycero-3-phosphatidylcholine | 2.14 | 1.34 | 0.00 | 808.58 | 90.19 |
| M468T195 | (M+H)+ | 1-Myristoyl-sn-glycero-3-phosphocholine | 5.03 | 2.66 | 0.00 | 468.31 | 194.98 |
| M457T244 | (M+Na-2H)- | 1-Oleoyl-L-.alpha.-lysophosphatidic acid | 1.18 | 1.61 | 0.00 | 457.24 | 244.15 |
| M522T188_2 | (M+H)+ | 1-Oleoyl-sn-glycero-3-phosphocholine | 5.20 | 1.09 | 0.00 | 522.35 | 187.87 |
| M454T198 | (M+H)+ | 1-Palmitoyl-2-hydroxy-sn-glycero-3-phosphoethanolamine | 1.40 | 1.80 | 0.00 | 454.29 | 198.02 |
| M496T191_3 | (M+H)+ | 1-Palmitoyl-sn-glycero-3-phosphocholine | 28.91 | 1.31 | 0.00 | 496.34 | 190.96 |
| M628T193 | (M+H-H2O)+ | 1-Stearoyl-2-arachidonoyl-sn-glycerol | 2.87 | 1.61 | 0.00 | 627.53 | 193.49 |
| M524T187_3 | (M+H)+ | 1-Stearoyl-2-hydroxy-sn-glycero-3-phosphocholine | 20.89 | 1.35 | 0.00 | 524.37 | 187.25 |
| M788T146 | M+ | 1-Stearoyl-2-oleoyl-sn-glycerol 3-phosphocholine (SOPC) | 4.93 | 1.14 | 0.03 | 787.60 | 145.59 |
| M70T315 | (M+H-2H2O)+ | Diethanolamine | 1.39 | 1.43 | 0.00 | 70.07 | 314.76 |
| M521T35 | (M+H-H2O)+ | N-Palmitoylsphingosine | 1.22 | 1.60 | 0.00 | 520.51 | 34.56 |
| M757T58_1 | (M+Na)+ | PC(16:0/16:0) | 1.84 | 1.37 | 0.00 | 756.55 | 58.45 |
| M169T56 | (M-H2O-H)- | 3-Hydroxycapric acid | 1.62 | 0.63 | 0.00 | 169.12 | 55.67 |
| M141T342_2 | (M-H2O-H)- | 2-Oxoadipic acid | 16.87 | 1.09 | 0.00 | 141.02 | 341.62 |
| M165T154 | (M+K-2H)- | Dihydrothymine | 1.99 | 0.76 | 0.00 | 165.00 | 153.59 |
| M267T216 | (M-H)- | Inosine | 1.04 | 0.16 | 0.00 | 267.07 | 215.78 |
| M243T162 | (M-H)- | Uridine | 1.15 | 0.84 | 0.00 | 243.06 | 162.18 |
| M120T260_2 | (M+H-H2O)+ | Tyramine | 2.16 | 1.14 | 0.01 | 120.08 | 259.69 |
| M137T290 | M+ | 1-Methylnicotinamide | 1.05 | 2.38 | 0.00 | 137.07 | 290.27 |
| M129T39 | (M-H)- | ketoisocaproic acid | 3.92 | 0.76 | 0.01 | 129.06 | 39.29 |
| M401T34_3 | (M+H)+ | 7-Oxocholesterol | 1.49 | 3.42 | 0.00 | 401.34 | 33.91 |
| M271T29 | (M+H-H2O)+ | Androstanedione | 2.48 | 0.08 | 0.00 | 271.21 | 28.53 |
| M411T33 | (M-H)- | hydropregnenolone sulfate | 2.45 | 0.21 | 0.00 | 411.18 | 33.16 |
| M395T82 | (M-H)- | pregnenolone sulfate | 0.97 | 0.35 | 0.00 | 395.19 | 82.05 |
| M165T79 | (M-H)- | 3-(2-Hydroxyphenyl)propionic acid | 1.42 | 4.18 | 0.00 | 165.06 | 79.24 |
