# Supplementary figures and images for: Untargeted LC/MS-Based Metabolic Phenotyping of Hypopituitarism in Young Males
Source: Front Pharmacol. 2021 Jul 8;12:684869. doi: 10.3389/fphar.2021.684869 (PMC8295757; doi:10.3389/fphar.2021.684869)

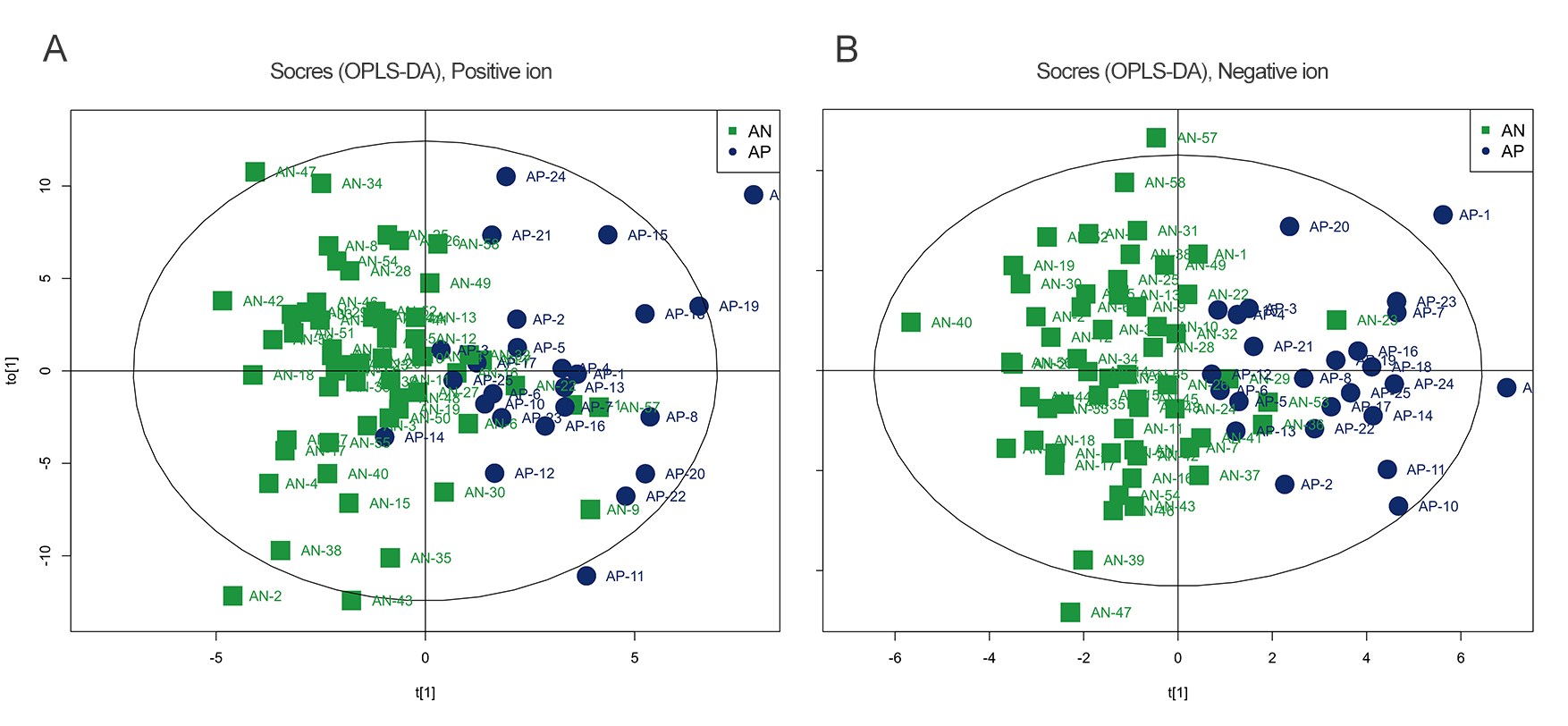

Supplement: Supplementary file 2 [file Image3.tif]

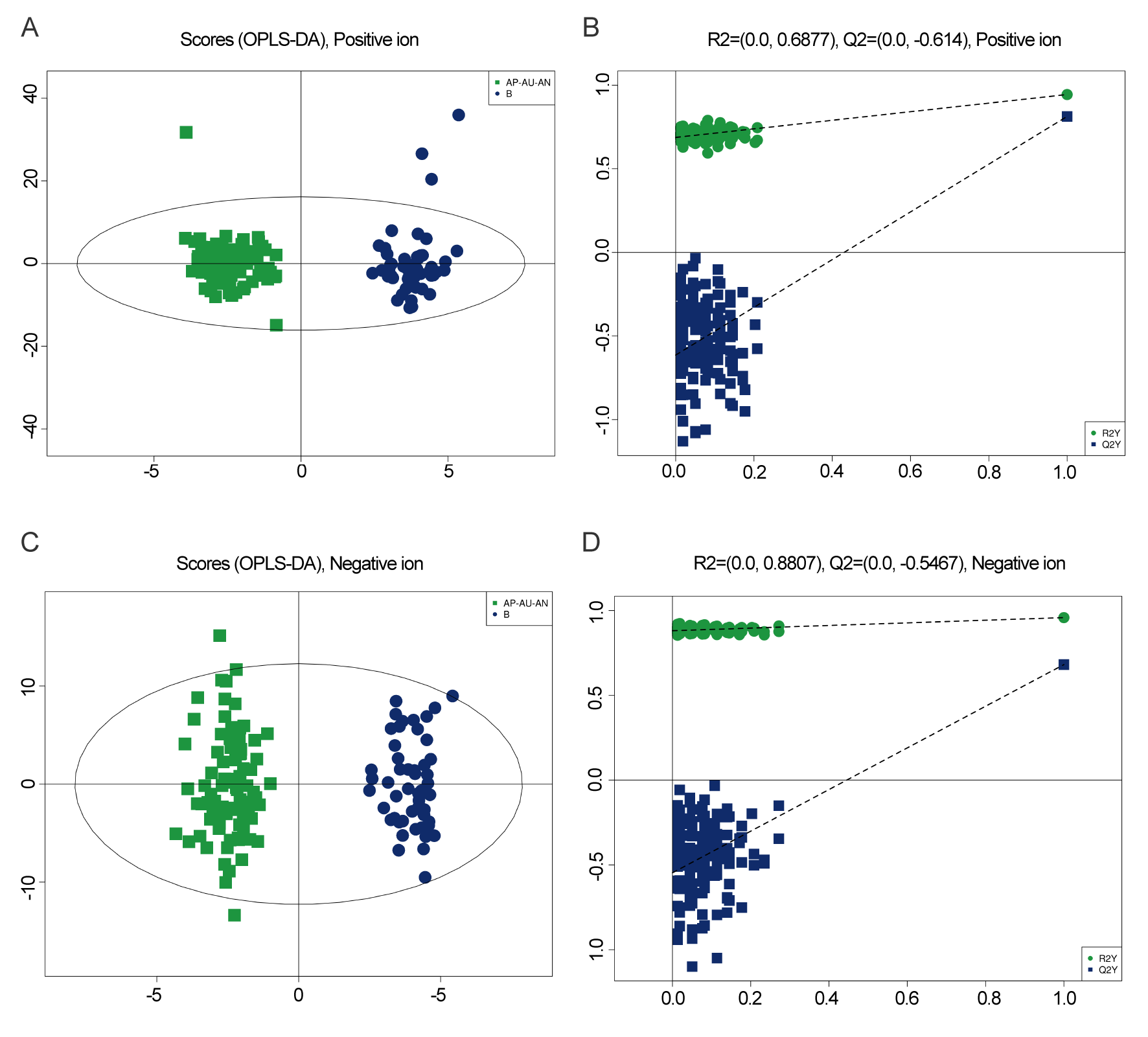

Supplement: Supplementary file 3 [file Image2.tif]

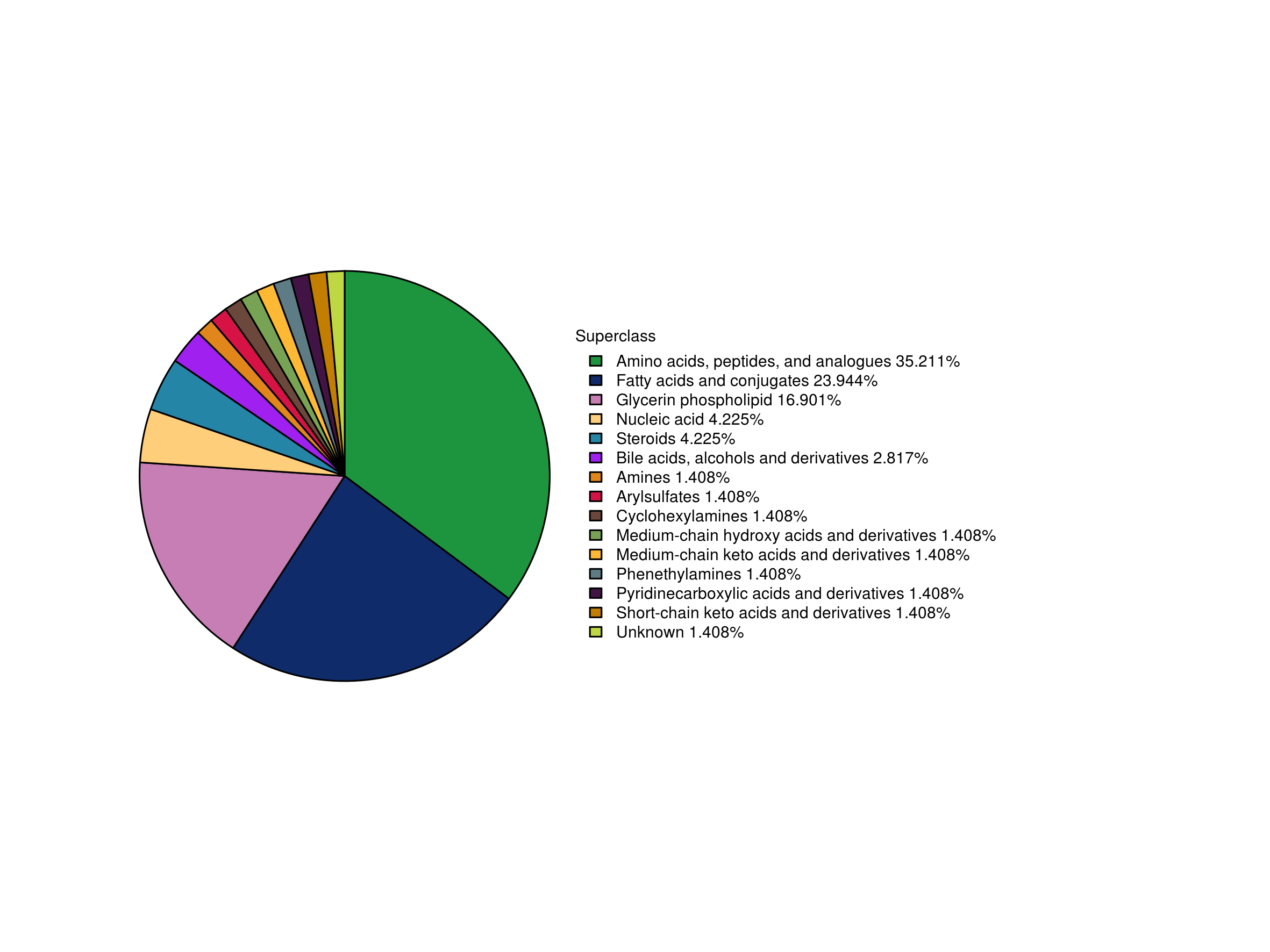

Supplement: Supplementary file 7 [file Image1.png]
