# Supplementary material for: Untargeted LC/MS-Based Metabolic Phenotyping of Hypopituitarism in Young Males
Source: Front Pharmacol. 2021 Jul 8;12:684869. doi: 10.3389/fphar.2021.684869 (PMC8295757; doi:10.3389/fphar.2021.684869)
Supplement: Supplementary file 4 [file Table2.docx]

**Supplement Table 2.** Differential metabolites between congenital and acquired Hypo-Pit

| **Name** | **Adduct** | **Description** | **VIP** | **Fold** | ***P*-value** | **m/z** | **rt(s)** |
| --- | --- | --- | --- | --- | --- | --- | --- |
| M759T59 | (M+Na)+ | Thioetheramide-PC | 32.23 | 1.20 | 0.03 | 758.57 | 59.20 |
| M279T46 | (M-H)- | Linoleic acid | 11.52 | 1.20 | 0.01 | 279.23 | 46.36 |
| M141T342_2 | (M-H2O-H)- | 2-Oxoadipic acid | 10.37 | 1.05 | 0.00 | 141.02 | 341.62 |
| M132T350_2 | (M+H)+ | Creatine | 8.20 | 2.46 | 0.00 | 132.08 | 350.02 |
| M212T32 | (M-H)- | Indoxyl sulfate | 7.87 | 1.32 | 0.04 | 212.00 | 31.96 |
| M114T170 | (M+H)+ | Creatinine | 7.37 | 0.90 | 0.01 | 114.07 | 170.48 |
| M524T187_3 | (M+H)+ | 1-Stearoyl-2-hydroxy-sn-glycero-3-phosphocholine | 5.77 | 1.06 | 0.03 | 524.37 | 187.25 |
| M209T35 | (M+H-H2O)+ | Myristoleic acid | 5.51 | 0.19 | 0.00 | 209.19 | 34.53 |
| M258T386 | M+ | Glycerophosphocholine | 4.96 | 0.76 | 0.00 | 258.11 | 386.15 |
| M124T294_2 | (M-H)- | Taurine | 4.13 | 1.17 | 0.00 | 124.01 | 294.33 |
| M130T262_2 | (M-H)- | L-Leucine | 3.30 | 1.20 | 0.01 | 130.09 | 261.97 |
| M277T48 | (M-H)- | all cis-(6,9,12)-Linolenic acid | 3.29 | 1.29 | 0.01 | 277.22 | 47.64 |
| M628T193 | (M+H-H2O)+ | 1-Stearoyl-2-arachidonoyl-sn-glycerol | 3.18 | 1.36 | 0.00 | 627.53 | 193.49 |
| M187T346 | (M-H)- | Azelaic acid | 3.14 | 1.48 | 0.03 | 187.10 | 345.86 |
| M116T298 | (M-H)- | L-Valine | 3.13 | 1.22 | 0.00 | 116.07 | 298.45 |
| M297T53_2 | (M-H)- | Nname,cis-9,10-Epoxystearic acid | 3.09 | 1.51 | 0.00 | 297.24 | 53.06 |
| M188T502 | (M+CH3CN+H)+ | L-Lysine | 2.98 | 0.59 | 0.00 | 188.14 | 502.13 |
| M164T255_2 | (M-H)- | L-Phenylalanine | 2.86 | 1.20 | 0.00 | 164.07 | 255.32 |
| M149T119 | (M-H)- | D-Lyxose | 2.68 | 0.81 | 0.03 | 149.04 | 119.21 |
| M184T89 | (M+H)+ | Phosphorylcholine | 2.54 | 1.47 | 0.00 | 184.07 | 89.33 |
| M165T79 | (M-H)- | 3-(2-Hydroxyphenyl)propionic acid | 2.49 | 3.21 | 0.03 | 165.06 | 79.24 |
| M90T350 | (M+H)+ | L-Alanine | 2.27 | 2.51 | 0.00 | 90.05 | 350.03 |
| M223T203 | (M+CH3COO)- | D-Quinovose | 2.24 | 1.25 | 0.00 | 223.08 | 203.17 |
| M203T256 | (M-H)- | L-Tryptophan | 2.20 | 1.27 | 0.00 | 203.08 | 256.45 |
| M118T303 | (M+H)+ | Betaine | 2.18 | 1.32 | 0.00 | 118.09 | 303.04 |
| M795T141 | (M+CH3CN+Na)+ | Sphingomyelin (d18:1/18:0) | 1.97 | 1.12 | 0.02 | 794.60 | 140.81 |
| M269T44 | (M-H)- | Heptadecanoic acid | 1.93 | 0.83 | 0.01 | 269.25 | 43.98 |
| M175T505_2 | (M+H)+ | L-Arginine | 1.71 | 1.07 | 0.02 | 175.12 | 505.38 |
| M583T62 | (M-H)- | Bilirubin | 1.66 | 1.80 | 0.00 | 583.25 | 62.24 |
| M163T203 | (M-H)- | L-Fucose | 1.62 | 1.23 | 0.00 | 163.06 | 203.26 |
| M391T123 | (M-H)- | Chenodeoxycholate | 1.60 | 1.25 | 0.01 | 391.28 | 122.77 |
| M496T213 | (M+H)+ | 1-Palmitoyl-sn-glycero-3-phosphocholine | 1.58 | 0.19 | 0.02 | 496.34 | 212.82 |
| M188T197 | (M+H-H2O)+ | DL-Indole-3-lactic acid | 1.57 | 1.81 | 0.00 | 188.07 | 196.58 |
| M338T34_3 | (M+H)+ | Erucamide | 1.53 | 1.11 | 0.02 | 338.34 | 33.88 |
| M174T496 | (M+CH3CN+H)+ | Ornithine | 1.51 | 0.57 | 0.00 | 174.12 | 496.20 |
| M104T386 | M+ | Choline | 1.43 | 0.79 | 0.00 | 104.11 | 386.15 |
| M154T399 | (M-H)- | L-Histidine | 1.43 | 1.25 | 0.01 | 154.06 | 399.41 |
| M161T301_2 | (M-H2O-H)- | D-Tagatose | 1.37 | 1.11 | 0.00 | 161.05 | 301.03 |
| M145T425 | (M-H)- | L-Glutamine | 1.33 | 2.24 | 0.00 | 145.06 | 425.34 |
| M130T400 | (M+H)+ | Pyroglutamic acid | 1.28 | 0.83 | 0.04 | 130.05 | 400.16 |
| M128T300_2 | (M-H)- | L-Pyroglutamic acid | 1.27 | 1.09 | 0.03 | 128.04 | 300.39 |
| M169T56 | (M-H2O-H)- | 3-Hydroxycapric acid | 1.27 | 1.23 | 0.00 | 169.12 | 55.67 |
| M337T32 | (M+H-H2O)+ | MG(18:2(9Z,12Z)/0:0/0:0)[rac] | 1.26 | 1.29 | 0.02 | 337.27 | 31.84 |
| M246T241 | M+ | 2-Methylbutyroylcarnitine | 1.23 | 1.24 | 0.02 | 246.17 | 240.59 |
| M295T49 | (M-H)- | 13(S)-HODE | 1.22 | 1.23 | 0.00 | 295.23 | 48.71 |
| M157T298 | (M+CH3CN+H)+ | D-Proline | 1.22 | 0.53 | 0.00 | 157.10 | 297.93 |
| M313T101 | (M-H)- | 9,10-DiHOME | 1.20 | 1.44 | 0.00 | 313.24 | 101.42 |
| M239T301 | (M+CH3COO)- | D-Mannose | 1.17 | 1.11 | 0.02 | 239.08 | 301.45 |
| M139T288 | (M-H2O-H)- | Allantoin | 1.13 | 1.10 | 0.05 | 139.03 | 287.96 |
| M137T290 | M+ | 1-Methylnicotinamide | 1.10 | 1.55 | 0.02 | 137.07 | 290.27 |
| M127T374 | (M-H)- | Dihydrothymine | 1.10 | 1.13 | 0.00 | 127.05 | 374.04 |
| M159T103 | (M-H)- | 3-Hydroxyvalproic acid | 1.06 | 1.30 | 0.01 | 159.10 | 102.51 |
| M84T400 | (M+H-H2O)+ | 1-Aminocyclopropanecarboxylic acid | 1.03 | 0.64 | 0.00 | 84.04 | 400.13 |
| M550T185_2 | M+ | 1-O-(cis-9-Octadecenyl)-2-O-acetyl-sn-glycero-3-phosphocholine | 1.03 | 1.19 | 0.00 | 550.38 | 185.41 |
| M281T128 | (M-H)- | Oleic acid | 1.02 | 1.12 | 0.04 | 281.25 | 127.82 |
| M267T101 | (M-H2O-H)- | Hexadecanedioic acid | 1.02 | 1.37 | 0.00 | 267.20 | 101.03 |
| M89T100 | (M-H)- | DL-lactate | 1.00 | 1.25 | 0.01 | 89.02 | 99.65 |
