# Supplementary material for: Untargeted LC/MS-Based Metabolic Phenotyping of Hypopituitarism in Young Males
Source: Front Pharmacol. 2021 Jul 8;12:684869. doi: 10.3389/fphar.2021.684869 (PMC8295757; doi:10.3389/fphar.2021.684869)
Supplement: Supplementary file 5 [file Table3.docx]

**Supplement Table 3.** Biomarkers for diagnostic of congenital Hypo-Pit vs. acquired Hypo-Pit

| Metabolites | (AN+AP) vs. B | | | | | |
| --- | --- | --- | --- | --- | --- | --- |
|  | AUC (95% CI) | Threshold | Sensitivity | Specificity | PPV | NPV |
| L-Alanine | 0.98 (0.96-0.98) | 6305 | 0.97 | 0.91 | 0.94 | 0.95 |
| Creatine | 0.95 (0.92-0.95) | 409836 | 0.84 | 0.94 | 0.95 | 0.78 |
| Creatine/Creatinine | 0.91 (0.85-0.91) | 0.14 | 0.84 | 0.90 | 0.93 | 0.78 |
| L-Lysine | 0.81 (0.73-0.81) | 60229 | 0.72 | 0.80 | 0.85 | 0.64 |
| L-Valine | 0.74 (0.65-0.74) | 270476 | 0.79 | 0.66 | 0.79 | 0.66 |
