# Supplementary material for: Untargeted LC/MS-Based Metabolic Phenotyping of Hypopituitarism in Young Males
Source: Front Pharmacol. 2021 Jul 8;12:684869. doi: 10.3389/fphar.2021.684869 (PMC8295757; doi:10.3389/fphar.2021.684869)
Supplement: Supplementary file 6 [file Table4.docx]

**Supplement Table 4.** Differential metabolites between hCG-sensitive and hCG-resistant Hypo-Pit

| **Name** | **Adduct** | **Description** | **VIP** | **Fold** | **P-value** | **m/z** | **rt(s)** |
| --- | --- | --- | --- | --- | --- | --- | --- |
| M70T315 | (M+H-2H2O)+ | Diethanolamine | 1.12 | 0.87 | 0.05 | 70.07 | 314.76 |
| M757T41 | (M+Na)+ | PC(16:0/16:0) | 1.52 | 0.85 | 0.05 | 756.55 | 41.21 |
| M316T188 | M+ | Decanoyl-L-carnitine | 1.05 | 0.80 | 0.05 | 316.25 | 187.87 |
| M132T267 | (M+H)+ | L-Leucine | 1.17 | 0.88 | 0.04 | 132.10 | 267.10 |
| M114T170 | (M+H)+ | Creatinine | 6.36 | 1.07 | 0.04 | 114.07 | 170.48 |
| M246T241 | M+ | 2-Methylbutyroylcarnitine | 2.05 | 0.80 | 0.04 | 246.17 | 240.59 |
| M241T49 | (M-H)- | Pentadecanoic Acid | 1.34 | 0.83 | 0.02 | 241.22 | 49.36 |
| M120T260_2 | (M+H-H2O)+ | Tyramine | 1.93 | 0.88 | 0.01 | 120.08 | 259.69 |
| M175T505_2 | (M+H)+ | L-Arginine | 1.31 | 0.92 | 0.01 | 175.12 | 505.38 |
| M162T355_2 | (M+H)+ | L-Carnitine | 4.70 | 0.92 | 0.01 | 162.11 | 354.84 |
| M496T191_3 | (M+H)+ | 1-Palmitoyl-sn-glycero-3-phosphocholine | 23.27 | 0.93 | 0.01 | 496.34 | 190.96 |
| M468T195 | (M+H)+ | 1-Myristoyl-sn-glycero-3-phosphocholine | 7.12 | 0.59 | 0.00 | 468.31 | 194.98 |
